# Factors Associated with Postoperative Skeletal Muscle Loss during Hospitalization in Patients with Non-Small Cell Lung Cancer Undergoing Lobectomy: An Exploratory Retrospective Cohort Study

**DOI:** 10.31662/jmaj.2025-0519

**Published:** 2026-04-24

**Authors:** Takashi Kido, Kengo Shirado, Kenta Kawamitsu, Shota Okuno, Rie Okamoto, Manabu Yasuda

**Affiliations:** 1 Department of Rehabilitation, Aso Iizuka Hospital Co., Ltd., Iizuka, Japan; 2 Department of Nutrition, Aso Iizuka Hospital Co., Ltd., Iizuka, Japan; 3 Department of Chest Surgery, Aso Iizuka Hospital Co., Ltd., Iizuka, Japan

**Keywords:** non-small cell lung cancer, skeletal muscle mass, postoperative skeletal muscle loss, perioperative care, perioperative rehabilitation

## Abstract

**Introduction:**

Postoperative skeletal muscle mass tends to decrease early, potentially affecting long-term prognosis and physical function. This study aimed to assess the extent of and factors associated with postoperative skeletal muscle loss (PSML) occurring during hospitalization in patients with non-small cell lung cancer (NSCLC) who were undergoing lobectomy.

**Methods:**

This retrospective cohort study included patients who underwent lobectomy for NSCLC. The extent of PSML during hospitalization―defined as the skeletal muscle mass index (SMI) loss rate [(preoperative SMI − SMI before discharge) / preoperative SMI × 100]―was evaluated along with associated factors, including patient background, rehabilitation assessments, surgical parameters, and postoperative course variables.

**Results:**

The cohort comprised 185 patients with a median age of 71.0 years (66.0-76.0), and 103 patients (55.7%) were male. The median SMI loss rate was 4.2%. Factors significantly associated with the SMI loss rate included female sex (B = 2.48; 95% confidence interval [CI]: 0.41-4.54; p = 0.018), body mass index (BMI) (B = 0.23; 95% CI: 0.07-0.38; p = 0.005), preoperative extracellular water-to-total body water ratio (ECW/TBW) (B = 223.80; 95% CI: 112.02-335.58; p < 0.001), difficulty completing three full meals per day by the fourth postoperative day (B = 1.69; 95% CI: 0.26-3.11; p = 0.020), and postoperative length of stay (B = 0.29; 95% CI: 0.06-0.52; p = 0.013).

**Conclusions:**

Patients with NSCLC who underwent lobectomy experienced a median PSML of 4.2% during hospitalization. PSML was more likely to occur in female patients and in patients with higher BMI, higher preoperative ECW/TBW, difficulty completing full meals by the fourth postoperative day, and longer postoperative length of stay. Preventive strategies against PSML in patients with NSCLC should emphasize perioperative management and rehabilitation tailored to individual patient characteristics.

## Introduction

The number of patients with cancer continues to increase worldwide ^(1)^. Surgical resection remains the standard curative treatment for many cancers and has contributed to improved survival ^(2)^. However, reports mainly from gastrointestinal surgery show that surgical procedures are often associated with a high incidence of postoperative skeletal muscle loss (PSML) ^(3), (4), (5), (6), (7), (8), (9)^. Recent studies have revealed that PSML is most significant during the early postoperative period, particularly during hospitalization ^(3), (4), (5), (6), (7), (8), (9)^. Increased PSML during this period is associated with poorer survival ^(3), (7), (9)^, lower quality of life ^(4)^, and decreased physical function ^(4)^. Therefore, implementing interventions to prevent PSML during hospitalization is essential for promoting early postoperative recovery in patients undergoing surgery.

Surgical resection is the standard treatment for stage I-II non-small cell lung cancer (NSCLC). In these patients, PSML may be a concern, similar to that observed in gastrointestinal surgery. Previous research has indicated that PSML occurring within one year postoperatively negatively impacts survival in patients with lung cancer ^(10)^. Consequently, early postoperative interventions to mitigate PSML are warranted in this population. However, to our knowledge, no studies have yet investigated the magnitude of PSML or its associated factors during the acute hospitalization period. Furthermore, perioperative care strategies and clinical guidelines differ substantially between gastrointestinal ^(11), (12)^ and thoracic surgery ^(13)^. Therefore, the risk factors for PSML identified in gastrointestinal surgery may not be directly extrapolated to patients undergoing lung cancer surgery.

This study, therefore, aimed to quantify PSML during the early postoperative period and to identify associated factors in patients undergoing lobectomy for NSCLC.

## Materials and Methods

### Study design

This retrospective cohort study included consecutive patients who underwent lobectomy for NSCLC at the Department of Chest Surgery of Iizuka Hospital (Iizuka-shi, Fukuoka, Japan) between April 2019 and June 2022.

The exclusion criteria were as follows: 1) stage IV lung cancer; 2) patients who required treatment for any disease other than postoperative complications during hospitalization; 3) patients who received chemotherapy during hospitalization; and 4) inability to undergo body composition analysis in the upright position or missing body composition data.

This study was approved by the Ethics Committee of Iizuka Hospital (approval number: 25004). An opt-out method was employed, which allowed patients to decline participation by reviewing the study information provided on the hospital’s website. This study was conducted in accordance with the Declaration of Helsinki, and patient protection was carefully considered.

### Perioperative care and rehabilitation

Perioperative care involves a comprehensive approach taken by a multidisciplinary management team that includes attending physicians, anesthesiologists, nurses, pharmacists, clinical engineers, dietitians, and physical therapists. Ventilator weaning was scheduled on the day of surgery, with oxygen therapy and chest drain management tailored to postoperative respiratory status. Pain control primarily relied on patient-controlled epidural or intravenous analgesia. If these were inadequate, multimodal analgesia was administered using nonsteroidal anti-inflammatory drugs and acetaminophen. Nutritional intake began on the first postoperative day with three meals per day. A registered dietitian provided meals to ensure a daily energy intake of at least 27 kcal/kg relative to standard body weight. Additionally, daily protein intake was adjusted to at least 1.0 g/kg in cases in which increased protein intake was deemed necessary based on the clinical condition or comorbidities. Ambulation was planned to commence within 24 hours after surgery, with rehabilitation beginning on the morning of postoperative day 1.

The rehabilitation adhered to a standardized protocol. Preoperatively, patients underwent body composition analysis, physical function testing, and perioperative orientation. Postoperatively, ambulation was initiated according to the hospital’s gait protocol, aimed at 30 m on the first day and 300 m on the second day. The patients underwent exercise therapy in the rehabilitation room from the third postoperative day until the day before discharge. These include strength training with equipment, aerobic exercises, functional training for daily activities, social reintegration, and lifestyle guidance. The discontinuation criteria followed the guidelines of the Japanese Association of Rehabilitation Medicine’s practice guidelines ^(14)^.

### Definition of PSML and body composition analysis

PSML was defined as a reduction in skeletal muscle mass occurring after surgery. In this study, we focused on PSML during hospitalization. Skeletal muscle mass was measured using bioelectrical impedance analysis (InBody 770; InBody, Seoul, Korea) before surgery and before discharge. Skeletal muscle mass was assessed using the skeletal muscle mass index (SMI; appendicular skeletal muscle mass [kg]/height[m]^2^). The SMI loss rate, calculated as [(Preoperative SMI − SMI before discharge) / Preoperative SMI × 100], was used to define PSML during hospitalization and served as the primary outcome variable. Patients whose SMI decreased compared with the preoperative value were classified into the PSML group, whereas those who maintained their SMI were classified into the non-PSML group. In addition, the extracellular water-to-total body water ratio (ECW/TBW) and phase angle (PhA) were recorded.

### Physical function assessment

Physical function was evaluated preoperatively and before discharge. The six-minute walk distance (6MWD) was measured according to the American Thoracic Society guidelines ^(15)^. Grip strength and usual walking speed were measured according to the recommendations of the Asian Working Group for Sarcopenia 2019 ^(16)^. For each measure, the change from the preoperative assessment to before discharge was calculated (Δ6MWD, Δgrip strength, and Δusual walking speed). Sarcopenia was diagnosed based on the Asian Working Group for Sarcopenia 2019 ^(16)^ criteria, including SMI, grip strength, and usual walking speed.

### Other variables

Data were collected from the medical records. The basic information included age, sex, and body mass index (BMI). Medical data included vital capacity (VC), percentage of VC, forced expiratory volume in 1 second (FEV1), FEV1/forced VC ratio, presence or absence of chronic obstructive pulmonary disease, comorbidities, cancer stage, Geriatric Nutritional Risk Index, and Brinkman index. Surgical data included the type of surgery, resection site, blood loss, operative time, anesthesia time, and lymph node dissection (LND). Postoperative factors included peak C-reactive protein levels, food intake through the fourth postoperative day, duration of chest tube drainage, postoperative complications, day of the first postoperative walk, day of walking independence, rehabilitation intervention time, and postoperative length of stay (LOS). The day of the first postoperative walking was defined as the number of days until the patient could walk at least 10 m ^(17)^. The day of walking independence was defined as the number of days until a physiotherapist judged that the patient was capable of performing ward activities independently.

New variables were derived from the collected data. The Charlson Comorbidity Index ^(18)^ was used to assess comorbidity risk, categorized as ≥2 or <2, based on the collected data ^(19)^. Postoperative food intake was categorized according to whether patients could complete three full meals per day by the fourth postoperative day ^(6), (7)^. Postoperative complications were classified using the Clavien-Dindo classification ^(20)^ as grade ≥Ⅱ or grade <Ⅱ.

### Statistical analysis

Continuous variables are presented as medians with interquartile ranges, and categorical variables as counts and percentages. As this study was designed as an exploratory observational study, we initially performed a univariable regression analysis using the SMI loss rate as the dependent variable to identify potential candidate variables. Variables with a p < 0.25 were considered candidate explanatory variables for the multivariable analysis ^(21)^. This relatively liberal threshold was adopted at the screening stage, as recommended in exploratory modeling, particularly in studies with a limited sample size or substantial variability, to reduce the risk of excluding variables that may be clinically important but not statistically significant in univariate analyses alone ^(21)^. Given the potential for multicollinearity among clinically related variables, one variable was selected as an explanatory variable for the multivariable analysis based on its clinical significance and interpretability, rather than solely on statistical criteria. Multivariable regression analysis was conducted to identify factors associated with the SMI loss rate. A complete case analysis with listwise deletion was used for statistical analysis. The normality of the residuals was visually assessed using quantile-quantile plots and verified using the Shapiro-Wilk test. Statistical significance was defined as p < 0.05. All statistical analyses were performed using EZR (Jichi Medical University, Tochigi, Japan), a graphical user interface for R (R Foundation for Statistical Computing, Vienna, Austria) ^(22)^.

## Results

### Participants and univariate analysis

**Figure 1.**
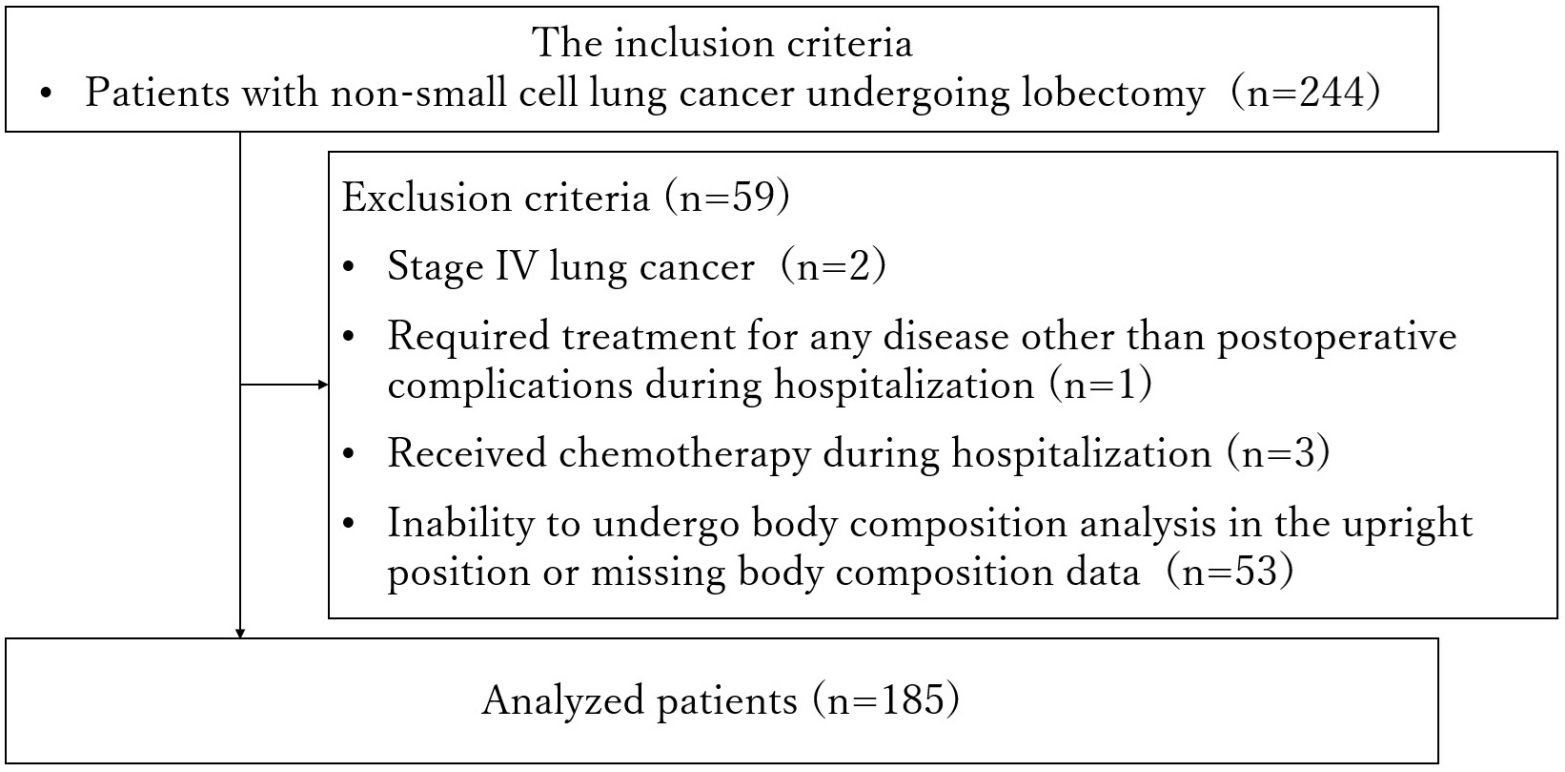
Patients’ flowchart.

Figure 1 shows the patient flowchart. A total of 244 patients met the eligibility criteria. A total of 185 patients were included in the analysis after excluding two patients with stage IV lung cancer, one patient who required treatment for a disease other than postoperative complications during hospitalization, three patients who received chemotherapy during hospitalization, and 53 patients who were unable to undergo body composition analysis in the upright position or had missing body composition data.

Continuous variables are presented as medians with interquartile ranges, and categorical variables as counts and percentages. Table 1 shows the baseline patient characteristics. The age of the cohort was 71.0 years (66.0-76.0), and 103 patients (55.7%) were male. The results of body composition analysis are presented in Table 2. Preoperative SMI was 6.5 kg/m^2^ (5.8-7.3), and SMI before discharge was 6.2 kg/m^2^ (5.5-7.0), representing a 4.2% (1.6-7.0) loss in SMI. Preoperative ECW/TBW was 0.392 (0.387-0.397), and ECW/TBW before discharge was 0.390 (0.385-0.395), indicating no perioperative increase in body water. Skeletal muscle mass by sex is as follows: Preoperative SMI was 7.1 kg/m^2^ (6.6-7.7) in male patients and 5.8 kg/m^2^ (5.3-6.3) in female patients. Before discharge, SMI was 6.9 kg/m^2^ (6.2-7.4) in male patients and 5.5 kg/m^2^ (5.1-5.9) in female patients. The loss in SMI was 3.8% (1.3-5.8) in male patients and 4.8% (3.2-7.6) in female patients, indicating that the loss in SMI was higher in female patients.

**Table 1.** Patient Characteristics.

Variable	Group	All	Missing data	Non-PSML group	PSML group
		n = 185		n = 33	n = 152
Basic property					
Age, years		71.0 [66.0 to 76.0]	0 (0)	70.0 [65.0 to 75.0]	71.0 [66.8 to 77.0]
Sex	Male	103 (55.7)	0 (0)	24 (72.7)	79 (52.0)
BMI, kg/m^2^		22.6 [19.9 to 25.6]	0 (0)	21.6 [18.3 to 24.7]	22.7 [20.1 to 25.6]
GNRI		105.0 [98.1 to 110.4]	0 (0)	104.6 [96.8 to 108.8]	105.5 [98.4 to 110.6]
CCI	≥2	14 (13.5)	0 (0)	4 (12.1)	21 (13.8)
COPD	Yes	14 (7.6)	0 (0)	5 (15.2)	9 (5.9)
Cancer stage	0	1 (0.5)	0 (0)	0 (0)	1 (0.7)
	Ⅰ	112 (60.5)		15 (45.5)	97 (63.8)
	Ⅱ	51 (27.6)		11 (33.3)	40 (26.3)
	Ⅲ	21 (11.4)		7 (21.2)	14 (9.2)
VC, L		2.9 [2.4 to 3.6]	0 (0)	3.3 [2.7 to 3.7]	2.8 [2.4 to 3.6]
%VC, %		99.7 [88.4 to 107.6]	0 (0)	102.4 [89.6 to 109.2]	98.2 [88.2 to 107.5]
FEV1, L		2.0 [1.7 to 2.5]	0 (0)	2.1 [1.7 to 2.8]	2.0 [1.7 to 2.4]
FEV1%, %		72.1 [67.0 to 77.7]	0 (0)	72.8 [64.8 to 77.8]	72.0 [67.8 to 77.7]
Brinkman Index		510.0 [0.0 to 920.0]	0 (0)	680.0 [285.0 to 940.0]	425.0 [0.0-893.8]
Preoperative rehabilitation assessment					
6MWD, m		437.0 [385.0 to 492.0]	6 (3.2)	445.0 [410.0 to 492.0]	436.0 [382.0 to 491.5]
Grip strength, kg		25.5 [20.7 to 33.6]	6 (3.2)	28.7 [22.5 to 33.4]	24.7 [20.7 to 33.5]
Usual walking speed, m/s		1.2 [1.0 to 1.3]	5 (2.7)	1.2 [1.1 to 1.2]	1.2 [1.0 to 1.3]
Sarcopenia	Yes	36 (20.1)	6 (3.2)	8 (25.0)	28 (19.0)
PhA, rad		4.6 [4.2 to 5.0]	0 (0)	4.7 [4.3 to 5.1]	4.6 [4.2 to 5.0]
Preoperative ECW/TBW		0.392 [0.387 to 0.397]	0 (0)	0.391 [0.385 to 0.395]	0.393 [0.387 to 0.398]
Operative factors					
Type of surgery	VATS	144 (77.8)	0 (0)	24 (72.7)	120 (78.9)
	Open chest	41 (22.2)		9 (27.3)	32 (21.1)
Resection site	Left upper lobe	38 (20.5)	0 (0)	11 (33.3)	27 (17.8)
	Left lower lobe	25 (13.5)		6 (18.2)	19 (12.5)
	Right upper lobe	69 (37.3)		8 (24.2)	61 (40.1)
	Right middle lobe	13 (7.0)		3 (9.1)	10 (6.6)
	Right lower lobe	40 (21.6)		5 (15.2)	35 (23.0)
Blood loss, mL		73.0 [30.0 to 175.0]	0 (0)	65.0 [30.0 to 140.0]	75.0 [30.0 to 201.3]
Operative time, min		208.0 [172.0 to 255.0]	0 (0)	210.0 [169.0 to 282.0]	208.0 [173.5 to 248.3]
Anesthesia time, min		305.0 [265.0-369.0]	0 (0)	295.0 [260.0 to 379.0]	305.5 [267.8 to 363.8]
LND	ND 1	28 (15.1)	0 (0)	5 (15.2)	23 (15.1)
	ND 2	157 (84.9)		28 (84.8)	129 (84.9)
Postoperative rehabilitation assessment					
Δ6MWD, m		-30.0 [-70.5 to -0.8]	33 (17.8)	-24.0 [-43.0 to 2.0]	-31.0 [-77.0 to -2.5]
Δgrip strength, kg		-1.0 [-2.2 to 0.5]	19 (10.2)	0.1 [-1.7 to 1.2]	-1.1 [-2.4 to 0.3]
Δusual walking speed, m/s		-0.1 [-0.2 to 0.1]	17 (9.1)	-0.1 [-0.1 to 0.1]	-0.1 [-0.2 to 0.1]
Factors related to the postoperative course					
Peak CRP		7.5 [5.4 to 12.0]	0 (0)	7.9 [6.3 to 11.1]	7.1 [5.3 to 12.0]
Clavien-Dindo classification	≥ Grade Ⅱ	24 (13.0)	0 (0)	3 (9.1)	21 (13.8)
Duration of chest tube drainage, days		2.0 [1.0 to 3.0]	0 (0)	1.0 [1.0 to 4.0]	2.0 [1.0 to 3.0]
Day of the first postoperative walk, day		1.0 [1.0 to 1.0]	0 (0)	1.0 [1.0 to 1.0]	1.0 [1.0 to 1.0]
Day of walking independence, day		3.0 [2.0 to 4.0]	0 (0)	3.0 [2.0 to 4.0]	3.0 [2.0 to 4.0]
Complete three meals a day by the fourth postoperative day	No	70 (37.8)	0 (0)	9 (27.3)	61 (40.1)
Rehabilitation intervention time, min		420.0 [300.0 to 560.0]	0 (0)	380.0 [220.0 to 460.0]	380.0 [280.0 to 520.0]
LOS, day		8.0 [7.0 to 11.0]	0 (0)	8.0 [6.0 to 11.0]	8.0 [7.0 to 11.3]

n (%), median [interquartile range]. 6MWD: Six-minute walking distance; BMI: body mass index; CCI: Charlson comorbidity index; COPD: chronic obstructive pulmonary disease; CRP: C-reactive protein; ECW/TBW: extracellular water-to-total body water ratio; FEV1: forced expiratory volume in 1 s; FEV1%: FEV1/forced VC ratio; GNRI: geriatric nutritional risk index; LND: lymph node dissection; LOS: postoperative length of stay; ND1: hilar node dissection; ND2: mediastinal node dissection; PhA: phase angle; PSML: postoperative skeletal muscle loss; VATS: video-assisted thoracic surgery; VC: vital capacity.

**Table 2.** Body composition analysis.

Variable	All	Male	Female
	n = 185	n = 103	n = 82
Preoperative SMI, kg/m^2^	6.5 [5.8-7.3]	7.1 [6.6-7.7]	5.8 [5.3-6.3]
SMI before discharge, kg/m^2^	6.2 [5.5-7.0]	6.9 [6.2-7.4]	5.5 [5.1-5.9]
SMI loss rate, %	4.2 [1.6-6.9]	3.8 [1.3-5.7]	4.8 [3.2-7.6]
Preoperative ECW/TBW	0.392 [0.387-0.397]	0.390 [0.386-0.395]	0.394 [0.389-0.398]
ECW/TBW before discharge	0.390 [0.385-0.395]	0.388 [0.384-0.394]	0.392 [0.387-0.397]

SMI loss rate, [(preoperative SMI − SMI before discharge) / preoperative SMI × 100]. n (%), median [interquartile range]. ECW/TBW: extracellular water-to-total body water ratio; SMI, skeletal muscle mass index.

Table 3 shows the results of univariable regression analysis of the occurrence of PSML. Variables with p < 0.25 included age, sex, BMI, VC, FEV1, grip strength, PhA, preoperative ECW/TBW, type of surgery, blood loss, operative time, anesthesia time, LND, Δ6MWD, Δusual walking speed, day of the first postoperative walk, Clavien-Dindo classification, completion of three full meals per day by the fourth postoperative day, intervention time, and LOS.

**Table 3.** Univariable Regression Analysis Regarding the Occurrence of PSML.

Variable	Group	All (n = 185)		
		Estimate	95% CI	p-Value
Basic property				
Age, years		0.08	0.01 to 0.14	0.024
Sex	Male	Reference		
	Female	1.70	0.47 to 2.92	0.007
BMI, kg/m^2^		0.15	-0.01 to 0.30	0.067
GNRI		0.04	-0.03 to 0.10	0.29
CCI	≥2	Reference		
	<2	-0.35	-2.17 to 1.46	0.69
COPD	Yes	Reference		
	No	1.04	-1.19 to 3.38	0.38
Cancer stage	0	Reference		
	Ⅰ	1.06	-7.27 to 9.40	0.81
	Ⅱ	0.19	-8.18 to 8.58	0.96
	Ⅲ	-1.77	-10.26 to 6.73	0.68
VC, L		-1.04	-1.86 to -0.23	0.012
%VC, %		0.02	-0.02 to 0.06	0.30
FEV1, L		-1.32	-2.38 to -0.26	0.015
FEV1%, %		-0.01	-0.06 to 0.06	0.94
Brinkman Index		-0.01	-0.01 to 0.01	0.76
Preoperative rehabilitation assessment				
6MWD, m		-0.01	-0.01 to 0.01	0.39
Grip strength, kg		-0.08	-0.15 to -0.01	0.026
Usual walking speed, m/s		-0.46	-2.81 to 1.89	0.69
Sarcopenia	No	Reference		
	Yes	-0.22	-1.78 to 1.35	0.79
PhA, rad		-0.59	-1.55 to 0.35	0.22
Preoperative ECW/TBW		157.17	77.80 to 236.53	<0.001
Operative factors				
Type of surgery	VATS	Reference		
	Open chest	-0.91	-2.39 to 0.59	0.23
Resection site	Left upper lobe	-1.05	-2.96 to 0.85	0.28
	Left lower lobe	-0.39	-2.54 to 1.75	0.71
	Right upper lobe	0.63	-1.03 to 2.31	0.45
	Right middle lobe	-0.64	-3.33 to 2.04	0.64
	Right lower lobe	Reference		
Blood loss, mL		-0.01	-0.01 to 0.01	0.183
Operative time, min		-0.01	-0.02 to 0.01	0.057
Anesthesia time, min		-0.01	-0.01 to 0.01	0.123
LND	ND 1	Reference		
	ND 2	-1.30	-3.02 to 0.42	0.137
Postoperative rehabilitation assessment			
Δ6MWD, m		-0.01	-0.02 to -0.01	0.028
Δgrip strength, kg		-0.14	-0.38 to 0.10	0.27
Δusual walking speed, m/s		-2.21	-4.95 to 0.53	0.114
Factors related to the postoperative course			
Peak CRP		0.01	-0.09 to 0.11	0.89
Clavien-Dindo classification	Grade <Ⅱ	Reference		
	Grade ≥Ⅱ	2.01	0.18 to 3.83	0.031
Duration of chest tube drainage, days		0.14	-0.10 to 0.37	0.26
Day of the first postoperative walk, day		0.45	-0.07 to 0.97	0.087
Day of walking independence, day		0.08	-0.15 to 0.32	0.48
Complete three meals a day by the fourth postoperative day	Yes	Reference		
	No	1.30	0.04 to 2.57	0.043
Rehabilitation intervention time, min		0.01	-0.01 to 0.01	0.20
LOS, day		0.26	0.11 to 0.40	<0.001

n (%), median [interquartile range]. 6MWD: Six-minute walking distance; BMI: body mass index; CI: confidence interval; CCI: Charlson comorbidity index; COPD: chronic obstructive pulmonary disease; CRP: C-reactive protein; ECW/TBW: extracellular water-to-total body water ratio; FEV1: forced expiratory volume in 1 s; FEV1%: FEV1/forced VC ratio; GNRI: geriatric nutritional risk index; LND: lymph node dissection; LOS: postoperative length of stay; ND1: hilar node dissection; ND2: mediastinal node dissection; PhA: phase angle; PSML: postoperative skeletal muscle loss; VATS: video-assisted thoracic surgery; VC: vital capacity.

### Variable selection and multivariable analysis

Considering clinical relevance, interpretability, and potential correlations among variables, the following variables were selected as explanatory variables for the multivariable regression analysis: age, sex, BMI, FEV1, grip strength, preoperative ECW/TBW, type of surgery, blood loss, operative time, LND, Δ6MWD, Δusual walking speed, day of the first postoperative walk, Clavien-Dindo classification, completion of three full meals per day by the fourth postoperative day, and LOS.

**Figure 2.**
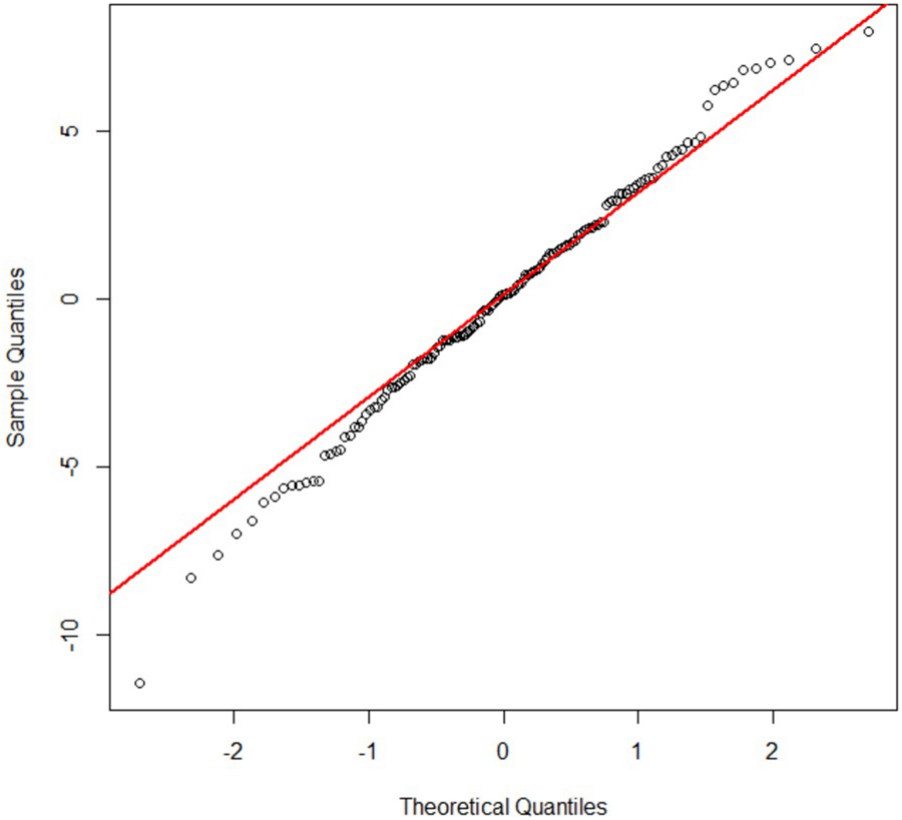
QQ plot of residuals. The residuals are approximately normally distributed, as indicated by their alignment along the reference line. QQ: quantile-quantile.

Table 4 shows the results of multivariable regression analysis for the occurrence of PSML. A statistically significant association with the SMI loss rate was found for female sex (B = 2.48; 95% confidence interval [CI]: 0.41-4.54; p = 0.018), BMI (B = 0.23; 95% CI: 0.07-0.38; p = 0.005), preoperative ECW/TBW (B = 223.80; 95% CI 112.02-335.58; p < 0.001), difficulty completing three full meals per day by the fourth postoperative day (B = 1.69; 95% CI: 0.26-3.11; p = 0.020), and LOS (B = 0.29; 95% CI: 0.06-0.52; p = 0.013). A quantile-quantile plot of the residuals is shown in Figure 2. The Shapiro-Wilk test results showed no statistically significant difference, and because normality was not rejected, the residuals were considered to follow a normal distribution (W = 0.99, p = 0.70).

**Table 4.** Multivariable Regression Analysis Regarding the Occurrence of PSML.

Variable	Estimate	95％ CI	p-Value
Age	-0.01	-0.09 to 0.09	0.92
Sex (female)	2.48	0.41 to 4.54	0.018
BMI	0.23	0.07 to 0.38	0.005
FEV1.0	0.57	-1.00 to 2.14	0.48
Grip strength	0.08	-0.04 to 0.19	0.21
Preoperative ECW/TBW	223.80	112.02 to 335.58	<0.001
Type of surgery (open chest)	-1.61	-3.50 to 0.29	0.096
Blood loss	-0.01	-0.01 to 0.01	0.52
Operative time	-0.01	-0.02 to 0.01	0.29
LND (ND2)	-0.73	-2.80 to 1.33	0.48
Δ6MWD	-0.01	-0.02 to 0.01	0.139
Δusual walking speed	0.25	-2.52 to 3.02	0.85
Days of first postoperative walking	0.64	-0.56 to 1.84	0.29
Clavien-Dindo Classification (grade ≥Ⅱ)	1.13	-1.21 to 3.48	0.34
Complete three meals a day by the fourth postoperative day (no)	1.69	0.26 to 3.11	0.020
LOS	0.29	0.06 to 0.52	0.013

6MWD: Six-minute walking distance; BMI: body mass index; CI: confidence interval; ECW/TBW: extracellular water-to-total body water ratio; FEV1: forced expiratory volume in 1 s; LND: lymph node dissection; LOS: postoperative length of stay; ND2: mediastinal node dissection; PSML: postoperative skeletal muscle loss.

## Discussion

We examined the factors associated with PSML during hospitalization in patients with NSCLC who underwent lobectomy and obtained two key findings. First, patients with NSCLC who underwent lobectomy experienced a PSML of 4.2% during hospitalization. Second, five factors were associated with PSML: sex, BMI, preoperative ECW/TBW, completion of three full meals per day by the fourth postoperative day, and LOS.

Patients with NSCLC who underwent lobectomy experienced a PSML of 4.2% during hospitalization. The observed loss in SMI is similar to the results seen in patients with gastrointestinal cancer, who experienced a 3.3%-4.4% loss during hospitalization ^(5), (9)^. Surgical stress triggers the release of inflammatory cytokines such as interleukin-6, which promotes corticosteroid secretion and increases insulin resistance, ultimately leading to protein breakdown ^(23), (24), (25)^. The transition from the catabolic phase to the anabolic phase following surgery typically takes approximately one week ^(24), (25)^. This indicates that the hospital stay is the period during which PSML associated with metabolic reactions is likely to occur. Although lung cancer differs from gastrointestinal cancer in terms of pathology, surgical technique, and perioperative management, these findings suggest that surgery-related metabolic responses induce a similar level of PSML regardless of cancer type. Previous studies in patients with lung cancer have shown that PSML occurring within one year after surgery is an important factor associated with survival, with an average loss of approximately 6% reported during this period ^(10)^. In this context, the present finding that PSML of 4.2% occurred within the relatively short hospitalization period indicates that a substantial proportion of PSML can develop over a highly compressed timeframe, underscoring the clinical importance of early PSML. Similarly, a previous study in patients with esophageal cancer reported that PSML during hospitalization was associated with poorer survival when the magnitude exceeded the median value of 4.4% ^(9)^. Taken together, these findings suggest that even a PSML of approximately 4% during hospitalization may be clinically meaningful and could potentially affect survival.

We identified five factors associated with PSML during hospitalization in patients with NSCLC undergoing lobectomy. First, a higher preoperative ECW/TBW was associated with a higher risk of PSML. Shiraishi and Ogawa ^(26)^ reported that among patients with femur fractures admitted to a convalescent ward, improvement in ECW/TBW during hospitalization was associated with an increase in SMI. Although our study assessed ECW/TBW at a single preoperative time point, it shares the common finding that ECW/TBW is associated with changes in skeletal muscle mass. ECW/TBW, the ratio of extracellular water to total body water, tends to be high in cases of malnutrition because the fluid shifts from the intracellular compartment to the extracellular compartment ^(27), (28)^. Compared with other nutritional indices, ECW/TBW has recently been shown to be a strong indicator of nutritional status ^(27), (28)^. It is also a sensitive marker of early fluid imbalance even before clinical edema ^(29)^. Kayano et al. ^(5)^ reported that preoperative malnutrition in patients with gastrointestinal cancer contributes to PSML. This finding indicates that preoperative malnutrition is associated with increased postoperative protein catabolism. In other words, preoperative ECW/TBW reflects preoperative nutritional status and may be associated with PSML by indicating the extent of postoperative protein catabolism.

Second, PSML was more likely to occur when patients were unable to complete three full meals per day by the fourth postoperative day. Hogenbirk et al. ^(7)^ found that patients with abdominal cancer who developed PSML had significantly lower nutritional intake by the fourth postoperative day than those without PSML. Our findings are consistent with those of previous studies on gastrointestinal surgery, highlighting the importance of early postoperative nutrition for patients undergoing lung cancer surgery. During hospitalization, energy expenditure increases markedly because of perioperative metabolic stress ^(24), (25)^, placing patients at a high risk of nutritional decline. Guidelines from the European Society for Clinical Nutrition and Metabolism ^(30)^ and the Enhanced Recovery After Surgery protocol ^(13)^ emphasize the importance of early oral feeding. Early enteral nutrition has been reported to reduce insulin resistance in patients undergoing gastric cancer surgery ^(31)^ and to lower the risk of persistent inflammation, immunosuppression, and catabolic syndrome in patients undergoing cardiac surgery ^(32)^. This indicates that sufficient nutritional intake during the early postoperative period helps to suppress the underlying factors of PSML. The digestive organs are not manipulated directly during lung cancer surgery, and oral intake typically resumes early. Despite the shortened postoperative fasting period in patients with lung cancer, nutritional intake may be impaired by postoperative nausea and vomiting, pain, or dyspnea. Individualized nutritional assessments and interventions are essential for patients who struggle to complete a full meal.

Third, female patients were more likely to develop PSML. Nagata et al. ^(33)^ reported that women were more likely to develop PSML after living donor liver transplantation, which supports our findings. Maintenance of skeletal muscle mass depends on the proper function of skeletal muscle stem cells ^(34)^. Estrogen, a hormone produced by the ovaries, plays a crucial role in this function ^(34)^. However, postmenopausal female patients often experience a decrease in estrogen levels due to decreased ovarian activity. This decline has been linked to a reduction in the number of skeletal muscle stem cells and an increase in apoptosis ^(34)^. These changes may predispose female patients to increased protein catabolism and PSML after surgery.

Fourth, PSML during hospitalization was more likely to occur in patients with high BMI. Surgical patients with obesity tend to have increased insulin resistance, prolonged systemic inflammation, and a higher risk of perioperative complications ^(35)^. Branched-chain amino acids ^(36)^ play a crucial role in promoting skeletal muscle anabolism and suppressing catabolism during the perioperative period. However, Biswas et al. ^(37)^ reported that patients undergoing cardiac surgery with high BMI were prone to postoperative metabolic abnormalities in branched-chain amino acids. This suggests that obesity may contribute to postoperative metabolic conditions associated with PSML. Takamori et al. ^(10)^ reported that BMI was not identified as a factor associated with PSML occurring approximately one year after lung cancer surgery, which differs from the findings of this study. This discrepancy is probably due to the fact that the study by Takamori et al. ^(10)^ focused on the maintenance phase, whereas our study targeted the acute phase, which resulted in different systemic conditions.

Fifth, a prolonged LOS was associated with a greater risk of PSML. Tanaka et al. ^(38)^ reported that the LOS was associated with PSML in patients with gastrointestinal cancer, supporting our findings. According to previous studies, prolonged LOS is negatively correlated with physical activity in patients undergoing thoracoabdominal and orthopedic surgeries ^(39)^. PSML has also been linked to reduced activity in patients undergoing abdominal surgery ^(7)^. Therefore, extended hospitalization may reflect or contribute to inactivity, thereby increasing the risk of PSML. Even after lung cancer surgery, interventions to maintain physical activity during hospitalization should be considered for patients who are expected to remain hospitalized for longer periods.

To our knowledge, this is the first study to evaluate the extent of PSML and its associated factors during hospitalization in patients with NSCLC undergoing lobectomy. These findings are clinically relevant and beneficial for improving the quality of care provided by multidisciplinary teams, including rehabilitation, nutrition, and nursing. Taken together, the findings for each variable indicate that the identified factors reflect interrelated aspects of patients’ baseline characteristics and perioperative conditions. However, formal causal inference was beyond the scope of this exploratory study. The potentially modifiable factors were BMI, preoperative ECW/TBW, and early postoperative meal intake. Previous studies suggested that BMI ^(40)^ and ECW/TBW ^(26)^ can be improved through rehabilitation, supporting the need to investigate preoperative rehabilitation in patients with lung cancer. As early postoperative intake is often impaired by postoperative nausea and vomiting ^(41)^, comprehensive perioperative interventions by the perioperative management team are necessary to ensure adequate nutrition and prevent PSML. Future studies should focus on these modifiable factors and test interventions using both retrospective and prospective designs. The 6MWD is a clinically relevant indicator of functional exercise capacity in patients with lung cancer ^(42)^. In the present study, univariable regression analysis showed a statistically significant association between PSML and a decline in the 6MWD. Although this relationship did not remain significant in multivariable regression analysis, this may reflect the short observation period during hospitalization, during which functional decline may not yet be fully detectable. These results suggest that early PSML may precede subsequent functional impairment, underscoring the need for longitudinal studies to clarify the temporal relationship between PSML and postoperative functional outcomes.

The limitations of this study are as follows: First, as this was a single-center retrospective study, there is a potential for bias in the study population. Patients for whom body composition analysis was not feasible or whose data were missing were excluded, which may have introduced selection bias and led to an underestimation of PSML. In addition, although previous studies in the field of gastrointestinal surgery have identified postoperative nutritional intake and physical activity levels ^(6), (7), (8)^ as important contributors to PSML, detailed measurements of these factors were not available in the present study. Consequently, unmeasured factors, such as detailed nutritional intake and physical activity levels, may have led to residual confounding. Future studies should more appropriately address these potential sources of bias. Second, the use of bioelectrical impedance analysis to measure skeletal muscle mass implies that fluctuations in perioperative total body water could have affected the impedance, potentially introducing measurement errors. This study demonstrated no significant changes in the body water balance, as evidenced by the ECW/TBW values before surgery and before discharge. Third, this study did not include data on long-term survival or patient-reported quality of life. Therefore, the clinical consequences of PSML beyond the hospitalization period could not be fully evaluated. Future prospective studies incorporating longitudinal outcomes and comprehensive functional assessments are warranted to clarify the broader impact of PSML.

### Conclusion

Patients with NSCLC who underwent lobectomy experienced a PSML of 4.2% during hospitalization. PSML was more likely to occur in patients with the following characteristics: female sex, high BMI, high preoperative ECW/TBW, difficulty completing three full meals per day by the fourth postoperative day, and prolonged LOS. Preventive strategies for PSML in patients with NSCLC undergoing lobectomy should focus on perioperative care and rehabilitation tailored to the patient’s background.

## Article Information

### Acknowledgments

We would like to thank Editage (www.editage.jp) for editing the English language.

### Author Contributions

Takashi Kido, Kengo Shirado, and Shota Okuno contributed to the study design and manuscript drafting. Kenta Kawamitsu contributed to data analysis and interpretation. Rie Okamoto and Manabu Yasuda contributed to data acquisition and critically revised the manuscript. All authors reviewed and approved the final version of the manuscript for submission.

### Conflicts of Interest

None

### IRB Approval Code and Name of the Institution

This study was approved by the Ethics Committee of Iizuka Hospital (approval No. 25004; February 17, 2025).

### Informed Consent

The requirement for individual informed consent was waived because of the retrospective design of the study and the use of an opt-out approach.
